# Sex and the Multidimensional Prognostic Index in 3.5-year post-COVID-19 mortality among older adults: evidence of a time-varying effect

**DOI:** 10.1007/s11739-025-04178-w

**Published:** 2025-11-07

**Authors:** Chiara Ceolin, Veronica Liberati, Margherita Vergadoro, Cristina Simonato, Sara Cazzavillan, Mario Virgilio Papa, Giulia Salerno Trapella, Benedetta Di Marzio, Bruno Micael Zanforlini, Chiara Curreri, Anna Bertocco, Giulia Gasparini, Maria Devita, Alessandra Coin, Luca Spiezia, Giuseppe Sergi, Marina De Rui

**Affiliations:** 1 Department of Medicine (DIMED), University of Padua, Via Giustiniani 2, 35128 Padua, Italy; 2 Geriatrics Division, University Hospital of Padua, Padua, Italy; 3 Department of Neurobiology, Care Sciences and Society, Karolinska Institutet and Stockholm University, Aging Research Center, Stockholm, Sweden; 4 First Chair of Internal Medicine, Padova University Hospital, Padua, Italy; 5 School of Community Medicine and Primary Health Care, University of Padua, Padua, Italy; 6 Department of General Psychology (DPG), University of Padua, Padua, Italy

**Keywords:** COVID-19, Multidimensional Prognostic Index, Frailty, Survival, Sex differences, Vaccination

## Abstract

**Purpose:**

The long-term prognostic impact of frailty in older adults recovering from COVID-19 remains underexplored. The Multidimensional Prognostic Index (MPI) has shown utility in predicting short-term outcomes, but its role over extended follow-up requires further investigation. The objective of this study is to evaluate the ability of an MPI-based model to predict 3.5-year mortality in older adults hospitalized for COVID-19.

**Methods:**

This single-center cohort study with prospective follow-up included 183 patients aged ≥ 65 years hospitalized with confirmed SARS-CoV-2 infection. MPI was calculated at admission and dichotomized into low (classes 1–2) and high (class 3). Multivariable Cox regression was used to estimate the hazard of mortality over a 3.5-year follow-up. Discriminative performance was assessed using time-dependent ROC analysis, with AUC values compared between the multivariable model and MPI alone.

**Results:**

During follow-up, 81/183 patients (44.3%) died. Kaplan–Meier curves showed lower survival in high-MPI patients (log-rank *p* = 0.0043). A Cox model with a time-varying effect for sex (sex × log(time)) confirmed that high MPI was associated with higher mortality (HR = 1.59, 95% CI 1.00–2.52), age was also associated (HR per year = 1.04, 95% CI 1.00–1.07), while vaccination was not. The female-to-male hazard ratio changed over time (HR at 180/365/730/1250 days: 0.90/1.31/1.87/2.48). AUCs for the full model at 180/365/730/1250 days were 0.704/0.654/0.680/0.659, derived from the sex-stratified Cox linear predictor, and exceeded the MPI-only model.

**Conclusions:**

The MPI demonstrated moderate prognostic ability for long-term mortality among older adults after COVID-19. Adding demographic and clinical variables modestly improved prediction, supporting the role of multidimensional assessment in geriatric prognosis, while highlighting the need for cautious interpretation over extended follow-up.

**Supplementary Information:**

The online version contains supplementary material available at 10.1007/s11739-025-04178-w.

## Introduction

The COVID-19 pandemic has disproportionately affected older adults, particularly those with frailty, a condition marked by reduced physiological reserve and impaired immune response [1, 2]. Frailty and multimorbidity have been consistently associated with higher mortality, prolonged recovery, and increased risk of functional decline after infection [3, 4]. Underlying mechanisms such as immunosenescence and chronic low-grade inflammation (“inflammaging”) contribute to a state of “homeostatic frailty,” which heightens vulnerability to infections and other complications commonly observed in hospitalized older patients [5, 6]. Despite some inconsistencies in the literature regarding the direct role of frailty in COVID-19 mortality, most evidence supports its relevance [1, 7]. Accurate identification of frailty is therefore essential, and comprehensive geriatric assessment (CGA) remains the gold standard for evaluating vulnerability and guiding clinical decisions in older patients with complex needs, including those affected by SARS-CoV-2 infection.

Among CGA-based frailty assessment tools, the Multidimensional Prognostic Index (MPI) has demonstrated strong prognostic value [8, 9]. Derived from CGA, the MPI captures multiple dimensions of health status—functional ability, cognitive performance, nutritional status, polypharmacy, comorbidity burden, and social vulnerability—integrating them into a single prognostic score. It has been extensively validated across various clinical populations, including cardiology [10], oncology [11], nephrology [12], and surgical cohorts [13], and is increasingly used to stratify mortality risk and inform therapeutic strategies [13, 14].

During the COVID-19 pandemic, however, relatively few studies have investigated the prognostic value of the MPI in older patients hospitalized with SARS-CoV-2 infection. Available evidence suggests that higher MPI scores—reflecting more advanced frailty—are associated with a greater burden of infections [5]. Other studies have reported that frailty, as measured by the MPI, is significantly associated with in-hospital mortality, prolonged length of stay, and reduced use of non-invasive ventilation, supporting the potential usefulness of the MPI in clinical decision-making during acute hospitalization for COVID-19 [8].

Nevertheless, a critical gap persists: no study to date has examined the long-term prognostic performance of the MPI beyond 1 year in this population. In particular, no previous research has assessed 3.5-year survival following COVID-related hospitalization in older adults. Addressing this knowledge gap is essential to understanding the enduring effects of frailty and to inform long-term care strategies in the post-COVID era. Moreover, emerging research has highlighted that both biological sex and sociocultural gender can influence the aging process, frailty progression, and immune response to infections such as SARS-CoV-2. Understanding how these variables intersect is crucial for improving personalized care and promoting equity in geriatric medicine.

This study aimed to evaluate the 3.5-year prognostic utility of an MPI-based model for predicting post-discharge mortality in older adults hospitalized with COVID-19.

## Methods

### Study population

The characteristics and recruitment criteria of the study population have been previously reported in detail [15–18]. Briefly, this analysis included a consecutive cohort of patients aged ≥ 65 years admitted to the Geriatrics Unit of the Azienda Ospedale—Università Padova with confirmed SARS-CoV-2 infection, regardless of the reason for hospitalization. Exclusion criteria included advanced dementia with inability to cooperate with clinical assessment, terminal illness with life expectancy < 1 month, active malignancy under treatment, immunosuppressive therapy (including high-dose corticosteroids or chemotherapy), and incomplete clinical or laboratory data at admission.

The study was conducted in accordance with Good Clinical Practice standards and the principles of the Declaration of Helsinki. The protocol was approved by the local ethics committee (Comitato Etico per la Sperimentazione Clinica della Provincia di Padova; protocol number 16412/AO/23), and written informed consent was obtained from all participants.

### Data collection

Detailed clinical, pharmacological, and functional characteristics of the study population have been previously reported [15–18]. In brief, data on comorbidities, and COVID-19-related information—including vaccination status, type and number of vaccine doses, and symptomatology—were extracted from medical records by experienced clinicians. Medication data, including total number of drugs, were also collected.

In addition, the following data were collected:

- *Multidimensional Prognostic Index (MPI)* The MPI is a prognostic index for 1-year mortality, calculated using information from the following scales: the Cumulative Illness Rating Scale (CIRS) for comorbidities [20], the Activities of Daily Living (ADL) [21] and Instrumental Activities of Daily Living (IADL) [22] for functional autonomy, the Mini Nutritional Assessment (MNA) [23] for nutritional status, the Short Portable Mental Status Questionnaire (SPMSQ) [24] for cognitive performance, and the Exton Smith scale (ESS) [25] for pressure sore risk. Additionally, data on the patient's medication regimen and cohabitation status were collected. The MPI score categorizes into three risk classes: class 1 (mild risk, score: 0–0.33), class 2 (moderate risk, score: 0.34–0.66), and class 3 (severe risk, score: 0.67–1.00).
- Follow-up for all-cause mortality was performed 3.5 years after hospital discharge.

### Statistical analysis

Continuous variables with normal distribution were expressed as means and standard deviations (SD), while those with non-normal distribution were reported as medians with interquartile ranges (IQR). The Shapiro–Wilk test was used to assess the normality of continuous variables. Categorical variables were presented as absolute counts and percentages. The Multidimensional Prognostic Index (MPI) was recoded into a binary variable: low MPI (classes 1 and 2) and high MPI (class 3). Group comparisons between survivors and non-survivors at 3.5-year follow-up were performed using independent samples t-tests or Mann–Whitney U tests for continuous variables, and Chi-square tests for categorical variables, as appropriate.

Survival analysis was conducted using Kaplan–Meier curves. Survival differences were evaluated first by MPI category (low vs. high), and subsequently by the interaction between MPI and sex (four groups). The log-rank test was used to compare survival distributions across groups. A Cox proportional hazards regression model was constructed to evaluate whether MPI was independently associated with 3.5-year mortality. Proportional hazards were assessed with Schoenfeld residual tests (cox.zph). Because PH was violated due to sex, we fitted an extended Cox model with a time-varying effect for sex by including sex × log(time) (implemented via tt(sex)); hazards for other covariates were assumed proportional. As a sensitivity analysis, we explored potential confounding by acute COVID-19 severity using the indicators available at admission (oxygen supplementation at Emergency Department arrival [L/min], presence of pneumonia, and ICU admission during the index hospitalization). Because these variables did not materially change the estimates for MPI or sex and did not improve model discrimination, and given the limited number of events, we retained a parsimonious final model including MPI, age, sex, and vaccination status to avoid overfitting.

We estimated time-dependent receiver operating characteristic (ROC) curves using the timeROC package in R. Areas under the curve (AUCs) were computed at 180, 365, 730, and 1250 days. For ROC estimation, to obtain a time-invariant linear predictor we used the sex-stratified Cox model. The linear predictor included MPI (High vs Low), age (years), and vaccination status (≥ 1 dose); sex was used as a stratification factor and was therefore not part of the linear predictor. As a comparator, we also computed AUCs for an MPI-only model. Results were similar when deriving the predictor from the extended Cox model with a time-varying effect of sex (sex × log(time)), but the stratified model was preferred for ROC to preserve a time-invariant predictor.

All statistical tests were two-tailed, and a *p* value < 0.05 was considered statistically significant. Survivors were administratively censored at 1250 days (end of follow-up). Analyses were conducted using IBM SPSS Statistics, version 29.0 (IBM Corp., Armonk, NY, USA) and R version 4.3.2 (R Core Team, Vienna, Austria).

## Results

### Sample characteristics

A total of 183 patients aged ≥ 65 years who were hospitalized for COVID-19 were included in the study. The mean age was 82.3 (SD = 12.9) years, and 48% were female. The majority (81.4%) were vaccinated at the time of hospital admission. During the 3.5-year follow-up, 81 patients (44.3%) died. Baseline characteristics stratified by survival status are reported in Table 1. Compared to survivors, patients who died during follow-up were older (mean age 83.4 ± 15.3 vs. 79.2 ± 10.4 years; *p* = 0.03), used a higher number of medications [median (IQR): 7 (5–9) vs. 4 (2–6.25); *p* < 0.001], and had higher MPI scores [0.63 (0.56–0.75) vs. 0.50 (0.32–0.69); *p* < 0.001]. In addition, they had a longer median hospital stay and a higher number of non-COVID-related hospitalizations. Vaccination status did not differ significantly between survivors and non-survivors.

**Table 1:** Characteristics of the sample according to survival status

Variable	All (n = 183)	Alive (n = 102)	Dead (n = 81)	*p* value
Age (years)	82.3 (12.9)	79.2 (10.4)	83.4 (15.3)	**0.03**
Sex F	88 (48.1%)	46 (45.1%)	42 (51.9%)	0.38
Vaccination (at least one dose)	149 (81.4%)	81 (79.4%)	68 (84.0%)	0.45
No. of drugs at admission	6 (3;8)	4.00 (2.00;6.25)	7.00 (5.00;9.00)	**< 0.001**
*Smoking habits (%)*				0.28
Active	12 (6.8%)	9 (9.1%)	3 (3.9%)	
Previous	48 (27.3%)	24 (24.2%)	24 (31.2%)	
*COVID-19 severity*				
O_2_ at admission (L/min)	0 (0;6)	0 (0;6)	0 (0;4.50)	0.75
Pneumonia at admission (%)	96 (56.5%)	55 (56.7%)	41 (56.2%)	0.94
Length of stay (days)	16.5 (11.25;25.00)	15 (11;23)	20 (12;28)	**0.04**
Intensive Care (%)	10 (6.1%)	9 (9.7%)	1 (1.4%)	**0.04**
Hospitalization unrelated to COVID (%)	72 (40.9%)	34 (33.74%)	38 (50.7%)	**0.03**
*Multidimensional geriatric assessment*				
ADL	1 (1;5)	2 (1;6)	1 (1;2)	** < 0.001**
IADL	1 (1;5)	3 (1;7)	1 (0;2)	** < 0.001**
MNA	18.50 (4.35)	19.25 (3.77)	16.22 (4.48)	** < 0.001**
CIRS-CI	5.00 (4.00;6.00)	5.00 (3.00;6.00)	5.50 (4.00;7.00)	**0.007**
ESS	13.50 (11.00;17.00)	15.00 (12.00;18.00)	12.00 (9.50;14.00)	** < 0.001**
SPMSQ	1 (0;5)	1 (0;4)	3 (1;7)	** < 0.001**
MPI	0.60 (0.44;0.71)	0.50 (0.32;0.69)	0.63 (0.56;0.75)	** < 0.001**

Values are expressed as means (standard deviation), medians (interquartile range) or counts (percentages %) as appropriate

F = females; O_2_ = Oxygen; ADL = Activities of Daily Living; IADL = Instrumental Activities of Daily Living; MNA = Mini Nutritional Assessment; CIRS-CI = Cumulative Illness Rating Scale—Comorbidity Index; SPMSQ = Short Portable Mental State Questionnaire; ESS = Exton Smith Scale; MPI = Multidimensional Prognostic Index

*p* values < 0.05 are reported in bold

### Survival analysis

**Fig. 1:**
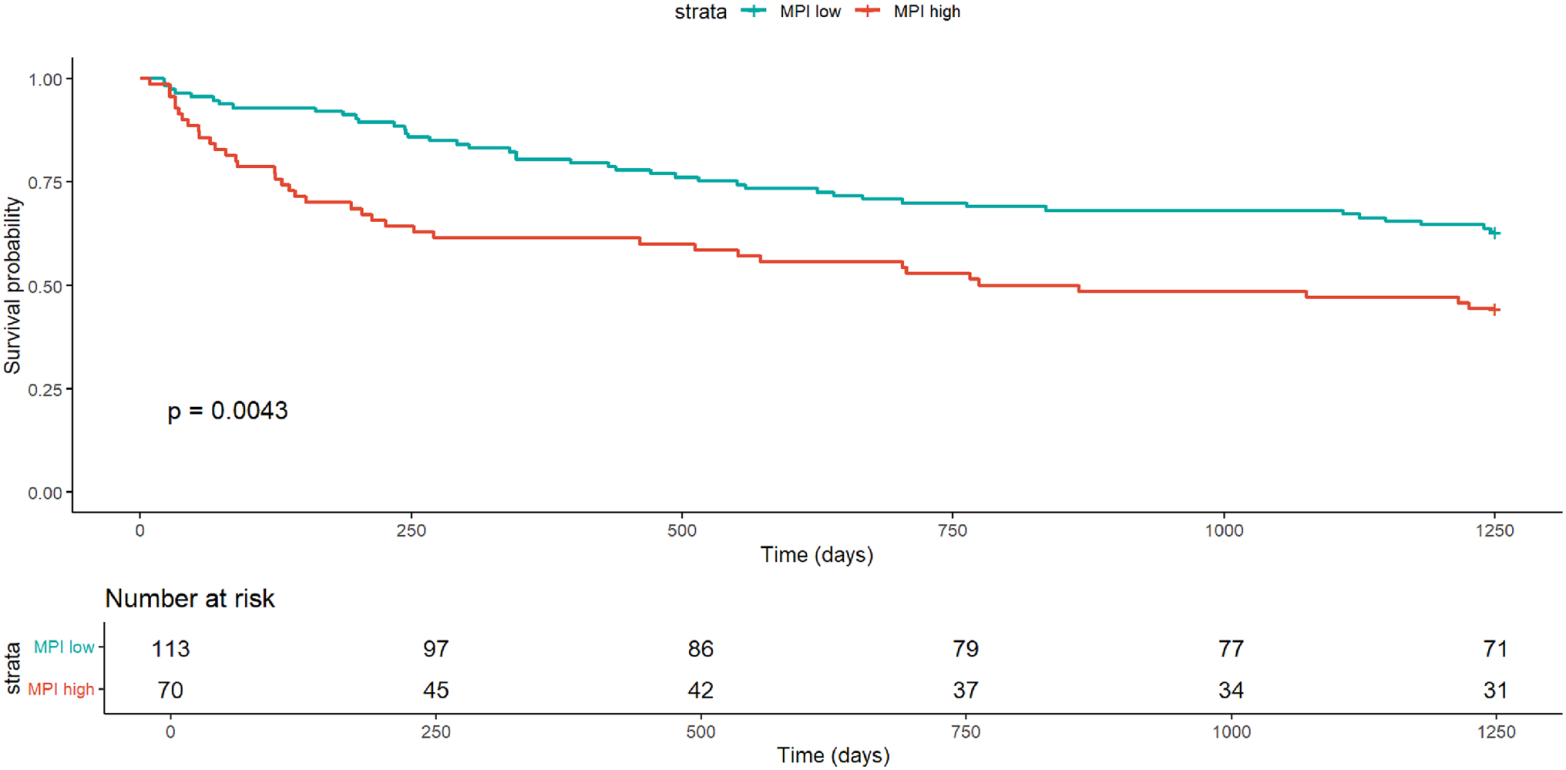
Kaplan–Meier survival curves stratified by MPI (low vs. high). Log-rank test *p* value = 0.0043. Survival curves are displayed up to 1250 days, corresponding to the maximum follow-up duration. Tick marks at key follow-up times (180, 365, 730, 1095, and 1250 days). Although no deaths occurred exactly at day 1250, this timepoint marks the end of follow-up (administrative censoring)

In the overall cohort, 113 patients (61.7%) were classified as having a low MPI and 70 (38.3%) as having a high MPI. Kaplan–Meier survival curves stratified by MPI category are shown in Fig. 1. A visible separation between the two groups emerged over time (*p* = 0.0043). Patients with a high MPI consistently showed lower survival probabilities compared to those with a low MPI, with the divergence emerging early after discharge and becoming more evident over time, suggesting a potentially meaningful prognostic gradient.

#### Sex-by-MPI survival and multivariable models

**Fig. 2:**
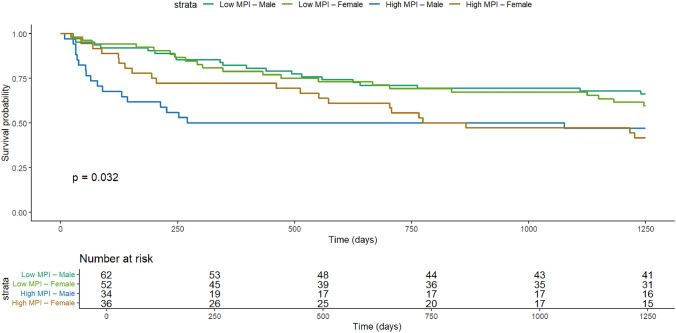
Kaplan–Meier survival curves stratified by sex and MPI classification. Survival curves are shown for four groups (Low MPI–Male, Low MPI–Female, High MPI–Male, High MPI–Female). Time is days from discharge; survivors were administratively censored at 1250 days. The curves differed overall (log-rank *p* = 0.032). KM curves include all participants with complete time-to-event and sex/MPI data (n = 184); Cox models use n = 183 due to missing covariates

Kaplan–Meier curves were stratified by sex and MPI (Fig. 2) and differed overall (log-rank *p* = 0.032). In Cox regression including a sex × MPI interaction, the interaction was not significant (Wald *p* = 0.303; likelihood-ratio *p* = 0.304), indicating no evidence that the effect of MPI differs by sex. Schoenfeld residuals revealed non-proportional hazards driven by sex (global *p* = 0.0032; sex *p* = 0.0083), with no evidence of PH violation for the MPI term. We therefore fitted an extended Cox model with a time-varying effect for sex (sex × log(time)). In this primary model, high MPI remained associated with higher mortality (HR = 1.59, 95% CI 1.00–2.52), independent of age (HR per year = 1.04, 95% CI 1.00–1.07) and vaccination (see Table 2, panel A for full estimates). The female-to-male hazard ratio varied over follow-up (HR at 180/365/730/1250 days: 0.90/1.31/1.87/2.48). As a sensitivity analysis, a sex-stratified Cox model yielded a very similar association for high versus low MPI (HR = 1.59, 95% CI 1.00–2.53; see Table 2, panel B).

**Table 2:** Multivariable Cox models for 3.5-year all-cause mortality (n = 183; events = 81)

(A) Primary model: extended Cox with time-varying effect for sex (sex × log(time))				
Variable	β (SE)	HR	95% CI	*p* value
High MPI versus low	0.463 (0.236)	1.59	1.00–2.52	0.050
Age (per year)	0.036 (0.017)	1.04	1.00–1.07	0.033
Vaccination (≥ 1 dose)	0.115 (0.306)	1.12	0.62–2.04	0.707
Female sex	− 2.813 (1.116)	0.06	0.007–0.54	0.012
Female × log(time)	0.522 (0.201)	1.69	1.14–2.50	0.009

(B) Sensitivity analysis: sex-stratified Cox model (strata(sex))				
High MPI versus low	0.467 (0.236)	1.59	1.00–2.53	0.048
Age (per year)	0.035 (0.017)	1.04	1.00–1.07	0.034
Vaccination (≥ 1 dose)	0.116 (0.306)	1.12	0.62–2.04	0.705

PH was violated due to sex; hence a time-varying effect for sex was modelled. The female-to-male hazard ratio at time t (days) is HR_Female/Male(t) = exp[β_Female + β_{Female × log(t)} · log(t)]. Representative HRs: 0.90 (180 d), 1.31 (365 d), 1.87 (730 d), 2.48 (1250 d). Models adjusted for age and vaccination; survivors administratively censored at 1250 days

### Prognostic accuracy

**Fig. 3:**
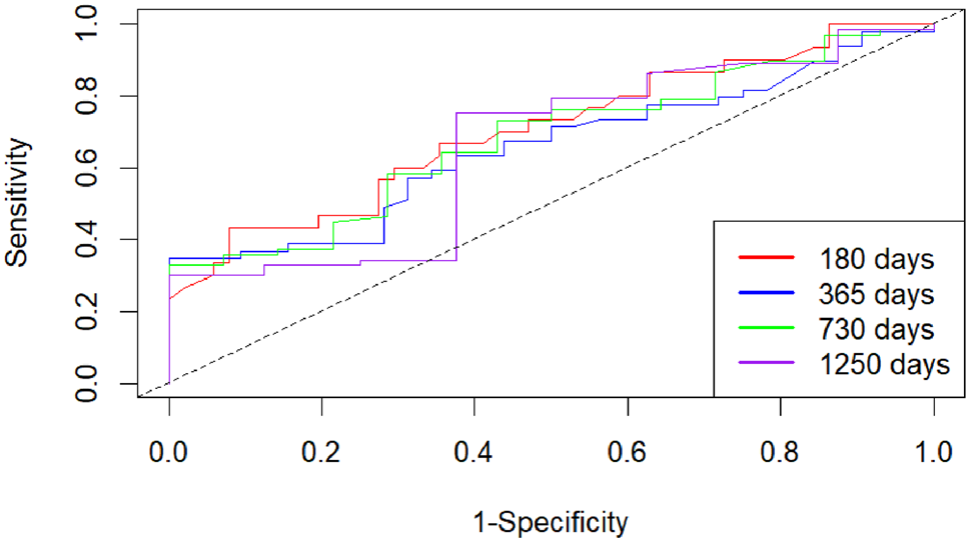
Time-dependent ROC curves of the prognostic model at 180, 365, 730, and 1250 days. AUC values were: 0.704 (95% CI 0.606–0.802), 0.654 (0.556–0.752), 0.680 (0.582–0.778), and 0.659 (0.561–0.757), respectively. The model demonstrated good short-term accuracy and moderate long-term discriminative ability. AUC values were derived from the sex-stratified Cox linear predictor (MPI, age, vaccination; sex used as stratification)

The discriminative performance of the multivariable model was assessed using time-dependent ROC analysis at 180, 365, 730, and 1250 days (Fig. 3). AUC values were derived from the sex-stratified Cox linear predictor (MPI, age, vaccination; sex used as stratification). The AUC values were 0.704 (95% CI 0.606–0.802) at 180 days, 0.654 (95% CI 0.556–0.752) at 365 days, 0.680 (95% CI 0.582–0.778) at 730 days, and 0.659 (95% CI 0.561–0.757) at 1250 days (3.5 years). The model demonstrated good predictive ability in the short term, with moderate discriminative performance maintained over time. At 3.5 years, the ROC curve appeared less smooth, likely due to a reduced number of patients remaining at risk.

### Comparison with MPI alone

To assess the added prognostic value of the multivariable model, we compared its discriminative performance to a model based solely on the MPI binary classification (Supplementary Figure 1). The AUC values for MPI alone were consistently lower across all time points: 0.674 (0.576;0.772) at 180 days, 0.588 (0.490;0.686) at 365 days, 0.532 (0.434;0.630) at 730 days, and 0.628 (0.530;0.726) at 1250 days. While the MPI alone provided an acceptable short-term risk estimate, its predictive capacity declined over time. In contrast, the multivariable model— incorporating age and vaccination (with sex used as a stratification factor)—maintained superior and more stable discriminative ability, especially in the medium- to long-term follow-up.

## Discussion

This study represents the first attempt to validate the MPI as a prognostic tool in a population of older adults hospitalized for SARS-CoV-2 infection, with a follow-up of 3.5 years after discharge. We found that high MPI was associated with higher 3.5-year mortality (HR = 1.59, 95% CI 1.00–2.52) after adjustment, and that the effect of sex was time-varying (male higher risk early, reversal later). Age showed a modest but significant association with mortality, while vaccination was not significantly associated in the long term. The multivariable model outperformed MPI alone across timepoints.

The prognostic value of the MPI has already been demonstrated in various clinical settings, particularly for short-term mortality. Previous studies on older patients with COVID-19 have linked higher MPI scores to increased in-hospital and ≤ 12 months mortality, typically focusing on the acute or early post-acute phases [8, 25, 26]. The current study extends these findings, providing novel evidence that MPI retains moderate but meaningful prognostic ability over a much longer follow-up (AUC ~ 0.66 at 1250 days). These results suggest that frailty at discharge reflects a biologically vulnerable phenotype with lasting implications, even as other health events accumulate over time. It should be noted, however, that the discriminative ability of the model was only moderate, with AUC values declining over time. This indicates that, while MPI and the multivariable model retain prognostic value, their accuracy in distinguishing survivors from non-survivors becomes more limited in the long term. Accordingly, long-term estimates should be interpreted with caution and validated in larger, multicenter cohorts.

The observed decline in MPI’s discriminative performance over time may be explained by several interrelated factors. First, the study examined all-cause mortality; deaths occurring beyond the first 12–18 months are more likely driven by unrelated conditions—such as cancer, cardiovascular disease, or accidents—not captured at baseline. Second, frailty is a dynamic, modifiable syndrome. Post-discharge interventions—including rehabilitation, nutritional optimization, or deprescribing—can improve frailty status and potentially lower risk [28, 29]. Conversely, lack of support or adverse events can accelerate decline [31]. Since the MPI was assessed only once at discharge, it does not account for such evolution, and additional unmeasured variables—like access to follow-up care or home-based services—may have influenced outcomes. Finally, the progressive reduction in the number of patients at risk over time decreased statistical power and limited the stability of long-term estimates.

A particularly notable finding was the role of female sex. Our modelling indicates a time-varying sex effect rather than a uniform protective association. Men had a higher early post-discharge risk, the female-to-male hazard ratio crossed neutrality around the first months, and later reversed. Hence, sex should not be interpreted as a constant protective factor; instead, its effect evolves over follow-up. These findings align with existing literature describing greater biological resilience in older women [32]. Women generally exhibit more favorable innate and adaptive immune responses and a lower pro-inflammatory milieu, potentially influenced by hormonal and genetic factors, which may mitigate severe post-infective sequelae and improve long-term resilience [33]. Several pathophysiological mechanisms may contribute. First, sex-steroid signaling (notably estrogen/ERα–ERβ) can enhance antiviral interferon responses, modulate B-cell class switching and antibody quality, and preserve endothelial function, whereas androgens have been linked to higher TMPRSS2 expression and pro-thrombotic tendencies [34–36]. Second, women typically show a slower trajectory of immunosenescence and lower inflammaging (e.g., lower IL-6/TNF-α tone), with better vaccine responsiveness and T-cell reserve, which may mitigate post-infective sequelae [37, 38]. Third, endothelial and coagulation biology tends to be more favorable in women, potentially buffering COVID-19–related endotheliopathy and downstream microvascular injury [39, 40]. Finally, metabolic and muscular resilience—including differences in mitochondrial bioenergetics, oxidative stress handling, and the trajectory of sarcopenia—may facilitate recovery from catabolic insults and reduce long-term vulnerability. While our study was not designed to dissect mechanisms, these pathways provide a biologically plausible explanation for the observed survival advantage and merit targeted investigation (e.g., longitudinal inflammatory panels, immune phenotyping, endothelial and coagulation markers). The interaction between sex and frailty underscores the importance of sex-aware prognostic models, particularly in vulnerable geriatric populations. While our analysis focused on sex differences, this study did not assess gender identity or psychosocial gender-related traits, which are increasingly recognized as modifiers of health outcomes. As noted by the literature, gender encompasses roles, norms, and behaviors that can influence exposure to risk, access to care, and treatment outcomes. The absence of these data is a limitation, and future studies should consider integrating validated gender measures to explore how biological and social dimensions interact to affect long-term outcomes in older adults post-COVID-19.

Surprisingly, vaccination—defined as having received at least one dose—was not significantly associated with long-term mortality, apparently contrasting earlier studies focused on short-term COVID-19 outcomes [41]. This result may reflect a saturation effect: over 80% of the cohort was vaccinated, likely reducing group contrasts. Moreover, late mortality is increasingly influenced by comorbidities and baseline frailty rather than the acute infection itself. While the protective effects of vaccination on COVID-19-related mortality are well established [17], our findings suggest that its long-term influence on all-cause mortality requires cautious interpretation and further research. Chronological age showed a modest but significant association with mortality in adjusted models, whereas frailty (MPI) provided additional prognostic information beyond age alone. This reinforces a foundational concept in geriatric medicine: age alone is a poor proxy for individual vulnerability. As shown by Rockwood et al. [42], frailty better predicts adverse outcomes than chronological age, capturing the multidimensional and heterogeneous nature of biological aging. Cesari et al. [43] further emphasized the need for clinical decision-making frameworks that prioritize person-centered assessments over rigid age thresholds. Such an approach promotes more equitable, precise, and effective care for older adults.

From a clinical perspective, these findings support the use of MPI at discharge for early identification of high-risk individuals. By incorporating modifiable factors such as function, cognition, and nutrition, the MPI can inform personalized care plans, including timely referral to rehabilitation or home-based services. Furthermore, integrating demographic variables like sex may improve risk stratification and optimize resource allocation. Adopting such models aligns with the broader goal of predictive, personalized, and proactive geriatric medicine.

### Limitations

Some limitations should be acknowledged. This was a single-center cohort study with prospective follow-up, with a homogeneous (Caucasian) population, which may limit generalizability. The relatively small sample (n = 183) and reduction in patients at risk over time constrained statistical power, particularly at later timepoints. In this regard, we recognize that the number of survivors decreased substantially after two years, which reduced the robustness of long-term estimates. Nevertheless, we decided to maintain the 3.5-year follow-up in order to provide information on the long-term prognostic role of frailty, while caution is warranted when interpreting results beyond 2 years. Furthermore, the MPI was calculated only at discharge, without longitudinal updates. Detailed physiological measures of respiratory failure (e.g., PaO_2_/FiO_2_ ratio, arterial blood gases, use and duration of non-invasive or invasive ventilation, organ-failure scores) were not systematically available. Therefore, although we tested proxy indicators of severity in sensitivity analyses, residual confounding due to acute disease severity remains possible. Additionally, all-cause mortality was used as the outcome without cause-specific stratification, preventing distinctions between COVID-related and unrelated deaths.

## Conclusions

In conclusion, this study suggests that the MPI retains some prognostic value for long-term mortality among older adults hospitalized with COVID-19, although its discriminative ability was only moderate and declined over time. The observed association between higher MPI scores and 3.5-year mortality—independent of age, sex (modelled as time-varying), and vaccination status—supports the role of multidimensional frailty as a relevant, though not definitive, determinant of long-term outcomes. Future research should aim to validate these findings in larger and more diverse populations, integrate dynamic frailty models, and include gender-related variables to better capture the complexity of individual vulnerability across biological and sociocultural domains.

## Supplementary Information

Below is the link to the electronic supplementary material.

Supplementary file1 (DOCX 25 kb)

## Data Availability

The datasets generated during and/or analysed during the current study are not publicly available due to privacy reasons but are available from the corresponding author on reasonable request.
