# Supplementary material for: Sex and the Multidimensional Prognostic Index in 3.5-year post-COVID-19 mortality among older adults: evidence of a time-varying effect
Source: Intern Emerg Med. 2025 Nov 7;21(2):459–67. doi: 10.1007/s11739-025-04178-w (PMC13061829; doi:10.1007/s11739-025-04178-w)
Supplement: Supplementary file 1 — Supplementary file1 (DOCX 25 kb) [file 11739_2025_4178_MOESM1_ESM.docx]

**What COVID-19 taught us about frailty: 3.5-year mortality prediction in older adults using the Multidimensional Prognostic Index-based model**

**Running head:** MPI and 3.5-year mortality after COVID-19 in older adults

Chiara Ceolin^1,2,3^*; Veronica Liberati^2^; Margherita Vergadoro^1,4,5^; Cristina Simonato^2*^; Sara Cazzavillan^2^; Mario Virgilio Papa^1^; Giulia Salerno Trapella^2^; Benedetta Di Marzio^2^; Bruno Micael Zanforlini^2^; Chiara Curreri^2^; Anna Bertocco^2^; Giulia Gasparini^2^; Maria Devita^2,6^; Alessandra Coin^1,2^; Luca Spiezia^1,4,5^; Giuseppe Sergi^1,2^; Marina De Rui^2^

*Corresponding author

^1^Department of Medicine (DIMED), University of Padua, Italy.

^2^Geriatrics Division, University Hospital of Padua, Padua, Italy

^3^Department of Neurobiology, Care Sciences and Society, Karolinska Institutet and Stockholm University, Aging Research Center, Stockholm, Sweden

^4^First Chair of Internal Medicine, Padova University Hospital, Italy

^5^School of Community Medicine and Primary Health Care, University of Padua, Italy

^6^Department of General Psychology (DPG), University of Padua, Italy

**Corresponding author**

Chiara Ceolin, Department of Medicine (DIMED), University of Padua, Via Giustiniani 2, 35128 Padua, Italy

Fax: +39 049 8211218; Phone: +39 049 8218949; E-mail: chiara.ceolin.1@studenti.unipd.it

**Supplementary Figure 1. Comparison of AUC values: multivariable model vs. MPI alone**


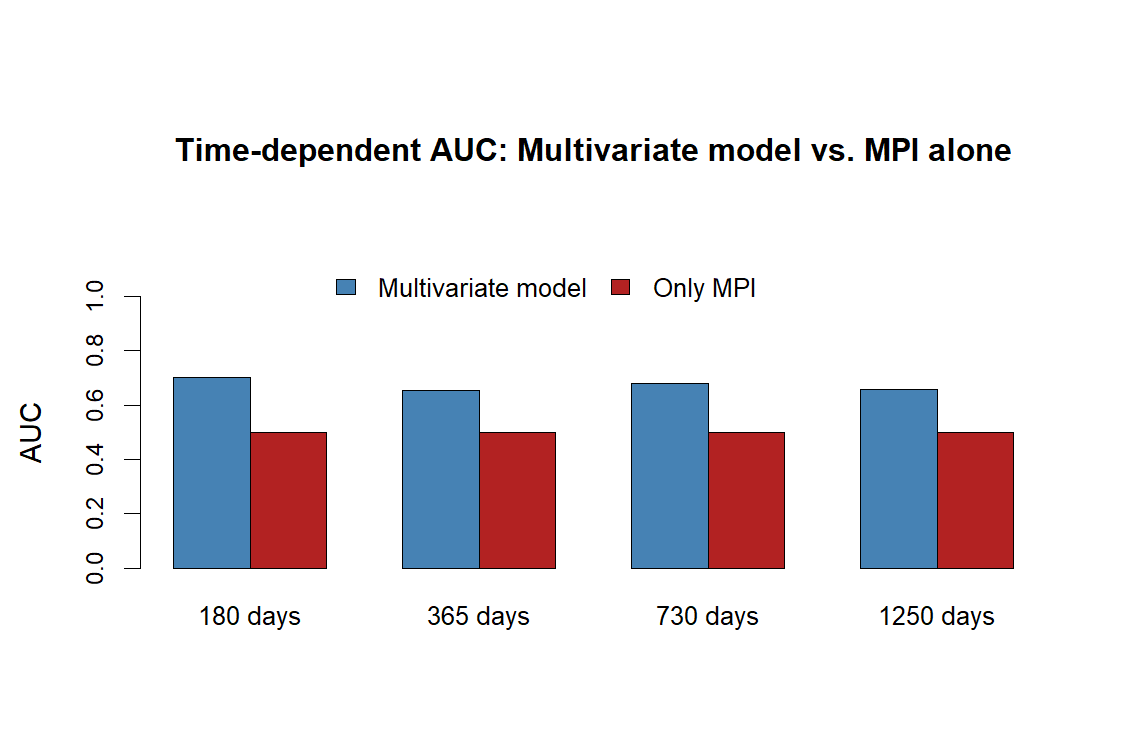


*Bar plot comparing AUC values and 95% confidence intervals for the multivariable model versus MPI alone. The multivariable model showed consistently higher discriminative performance at all time points.*
